# *Streptococcus thermophilus* APC151 Strain Is Suitable for the Manufacture of Naturally GABA-Enriched Bioactive Yogurt

**DOI:** 10.3389/fmicb.2016.01876

**Published:** 2016-11-23

**Authors:** Daniel M. Linares, Tom F. O’Callaghan, Paula M. O’Connor, R. P. Ross, Catherine Stanton

**Affiliations:** ^1^Food Biosciences Department, Teagasc Food Research Centre MooreparkFermoy, Ireland; ^2^APC Microbiome Institute, University College CorkCork, Ireland

**Keywords:** GABA, *Streptococcus thermophilus*, bioactive yogurt, starter, biofunctional food

## Abstract

Consumer interest in health-promoting food products is a major driving force for the increasing global demand of functional (probiotic) dairy foods. Yogurt is considered the ideal medium for delivery of beneficial functional ingredients. Gamma-amino-butyric acid has potential as a bioactive ingredient in functional foods due to its health-promoting properties as an anti-stress, anti-hypertensive, and anti-diabetic agent. Here, we report the use of a novel *Streptococcus thermophilus* strain, isolated from the digestive tract of fish, for production of yogurt naturally enriched with 2 mg/ml of gamma-amino-butyric acid (200 mg in a standard yogurt volume of 100 ml), a dose in the same range as that provided by some commercially available gamma-amino-butyric acid supplements. The biotechnological suitability of this strain for industrial production of yogurt was demonstrated by comparison with the reference yogurt inoculated with the commercial CH1 starter (Chr. Hansen) widely used in the dairy industry. Both yogurts showed comparable pH curves [ΔpH/Δ*t* = 0.31-0.33 h^-1^], viscosity [0.49 Pa-s], water holding capacity [72–73%], and chemical composition [moisture (87–88%), protein (5.05–5.65%), fat (0.12–0.15%), sugar (4.8–5.8%), and ash (0.74–1.2%)]. Gamma-amino-butyric acid was not detected in the control yogurt. In conclusion, the *S. thermophilus* APC151 strain reported here provides a natural means for fortification of yogurt with gamma-amino-butyric acid.

## Introduction

*Streptococcus thermophilus* is a non-pathogenic and homofermentative facultative anaerobic lactic acid (LAB) bacterium with a long history of use in the home-made and modern industrial manufacture of fermented dairy products, especially yogurt (Wu et al., 2014). As a dairy starter, *S. thermophilus* can rapidly convert lactose into lactic acid, which causes a rapid reduction in pH resulting in coagulation of milk proteins (casein). In addition, this bacterium confers many excellent processing properties to the yogurt, such as flavor, acidity, viscosity, and water holding capacity (Sun et al., 2011).

Since yogurt contains live cultures, it has been considered an ideal vehicle to deliver probiotic and biofunctional ingredients to the gut. Beneficial effects can be provided by classic strains associated with the technological production of the yogurt (*S. thermophilus* and *Lactobacillus delbrueckii* subsp. *bulgaricus*), or by additional strains supplemented in order to provide a probiotic function (generally *Lactobacillus* or *Bifidobacterium*). In this regard, yogurt bacteria have been shown to have a favorable impact on digestive health improving lactose digestion in lactose-intolerant individuals (Rul et al., 2011; Savaiano, 2014), increasing intestinal regularity and digestion (Fioramonti et al., 2003), preventing diarrhea (Beniwal et al., 2003; Linares et al., 2016) and inflammatory bowel disease (Lorea Baroja et al., 2007; Shadnoush et al., 2013), and stimulating the gut immune system (Hong et al., 2015; Maldonado-Galdeano et al., 2015).

Discovered in 1950, gamma-aminobutyric acid (GABA) is the main inhibitory neurotransmitter naturally occurring in the central nervous system (CNS) and some peripheral tissues (Bravo et al., 2011). Several important physiological functions of GABA have been characterized, such as neurotransmission, diuretic effects, relaxing, and tranquilizer effects (Hayakawa et al., 2004; Li and Cao, 2010). In fact, some GABA_A_ (a type of GABA receptors) agonist drugs are important pharmacological agents used for clinical treatment of anxiety (e.g., benzodiazepines) (Foster and Kemp, 2006). In addition to this stress management role, GABA was reported to lower blood pressure in humans with mild hypertension (Shimada et al., 2009; Pouliot-Mathieu et al., 2013) and to have a significant anti-diabetic effect (Adeghate and Ponery, 2002; Wan et al., 2015). Beside this, patients with Alzheimer’s disease have a decreased level of GABA in their temporal cortex, occipital cortex and cerebellum (Seidl et al., 2001). Thus, GABA has been classified as a health-promoting bioactive component in foods and pharmaceuticals (Li and Cao, 2010). In fact, foods enriched with GABA are defined as FOSHU (Foods for Specified Health Use) by the Japanese government (Rizzello et al., 2008). A number of commercial sources sell formulations of GABA for use as a dietary supplement claiming to have a calming and tranquilizing effect. One of them, Pharma-GABA^TM^, a natural form of GABA has been approved by the FDA as a food ingredient (Food and Drug Administration [FDA], 2008).

Several lactic acid bacteria (LAB) have been reported to exhibit GABA-producing ability through α-decarboxylation of glutamate, an enzymatic conversion which is catalyzed by glutamate decarboxylase (GAD) (Tajabadi et al., 2015). Among them, most of the GABA-producing strains are lactobacilli (mainly *Lb. brevis, Lb. paracasei, Lb. delbrueckii, Lb. buchneri, Lb. plantarum*, and *Lb. helveticus*) and *Lactococcus lactis* (Li and Cao, 2010; Dhakal et al., 2012; Wu et al., 2015). Alternatively, *Bifidobacterium* spp strains were reported to produce GABA, although they have a low capacity for production (Park et al., 2005; Barrett et al., 2012). So far, only a few *S. thermophilus* strains were reported to produce significant levels of GABA from glutamate (Yang et al., 2008). However, the suitability of these strains to produce GABA in fermented milk and yogurt has not yet been explored. The ability of LAB to biosynthesize GABA is not present in all strains belonging to one species, as it is a strain-dependent attribute genetically associated to the genes *gadB* and *gadC* (Tajabadi et al., 2015). In addition, even among those GAD^+^ strains belonging to the same species, the efficiency and yield of GABA production varies markedly (Dhakal et al., 2012). The capacity to produce GABA in high yields is rare among conventional *S. thermophilus* (generally from dairy origin), thus we screened less explored sources such as the gastrointestinal tract (GIT) of fish (*Labrus mixtus*) caught off the coast of Ireland.

Since *S. thermophilus* is a commercially relevant starter widely exploited for the industrial production of yogurt, and GABA has a potential as bioactive component in foods and pharmaceuticals (Li and Cao, 2010; Wu and Shah, 2016), in this work, we tested the biotechnological suitability of the newly isolated strain to coagulate milk and produce naturally GABA-enriched yogurt. The quality and physical properties of the obtained GABA-rich yogurt were compared with those of reference yogurts produced in parallel under similar conditions using commercial starters widely used in the dairy industry.

## Materials and Methods

### Bacterial Culture Medium and Conditions

*Streptococcus thermophilus* strains were grown at 42°C in M17 medium (Oxoid, Basingstoke, UK) supplemented with 0.5% lactose (LM17). *L. delbrueckii* subsp. *bulgaricus* strains were anaerobically grown in MRS (Difco, Detroit, MI, USA). Unless indicated otherwise, the medium was supplemented with 2.25 mg/ml of monosodium glutamate (MSG; Sigma–Aldrich). When solid medium was required, 2% (w/v) agar (Oxoid, Hampshire, UK) was added to the corresponding medium.

### Isolation of GABA-Producer *S. thermophilus* Strain

*Streptococcus thermophilus* is the universal starter for yogurt fermentation. In order to isolate GABA-producer strains technologically suitable to produce yogurt, we carried out a screening in LM17. Because the capacity to produce GABA in high yields is rare among *S. thermophilus*, we screened less explored sources such as fish intestines. A *S. thermophilus* APC151 was isolated from the digestive tract of fish (*L. mixtus*) caught off the coast of Ireland. Briefly, the intestine was aseptically extracted, and content transferred into a sterile stomacher bag, diluted with sterile maximum recovery diluent (MRD; Oxoid Ltd., Basingstoke, UK) and homogenized for 3 min in a Stomacher 400 laboratory blender (Seward Ltd., London, UK). Further, serial dilutions were carried out in MRD, plated on LM17 agar plates and incubated anaerobically at 42°C.

### Detection and Quantification of GABA Production

Extracellular GABA that accumulated in the culture medium or yogurt was quantified by post-column ninhydrin derivatization on cation-exchange HPLC as follows. Samples of culture supernatants or yogurt were deproteinized by mixing equal volumes of 24% (w/v) trichloroacetic acid (TCA) and sample, these were allowed to stand for 10 min before centrifuging at 14400 × *g* (Microcentaur, MSE, UK) for 10 min. Supernatants were transferred to a new tube and diluted with 0.2 M sodium citrate buffer, pH 2.2, to give approximately 250 nmol of each amino acid residue. Samples were then diluted one in two with the internal standard, norleucine, yielding a final concentration of 125 nm/ml. GABA was quantified using a Jeol JLC-500/V amino acid analyser [Jeol (UK) Ltd., Garden city, Herts, UK] fitted with a Jeol Na^+^ high performance cation exchange column (Mounier et al., 2007).

### Yogurt Manufacture

The fermentation substrate consisted of 14% (w/v) skim milk (Kerry ingredients Ltd, Kerry, Ireland). For the elaboration of both yogurts, milk was previously supplemented with 2.25 mg/ml MSG. Prior to inoculation, milk was heat treated at 121°C for 5 min and then cooled to 42°C. The thermophilic starter CH1 (obtained from Chr. Hansen, Denmark) was used as reference for the production of plain set-style yogurt. The two strains composing this starter (*S. thermophilus* CH1 and *Lb. delbrueckii* subsp. *bulgaricus* CH1) were isolated and inoculated in milk in a 1:1 ratio (10^6^ cells of each). The GABA-enriched yogurt was manufactured in parallel under identical conditions as the control yogurt except that strain *S. thermophilus* CH1 was replaced by the GABA-producing strain *S. thermophilus* APC151. Thus, both yogurts differed only in the *S. thermophilus* strain. Following inoculation, yogurts were fermented in 100 ml cups at 42°C for 48 h, and then the samples were rapidly cooled to 4°C and stored at this temperature for up to 3 weeks. GABA content, viable bacteria counts, pH, viscosity, water holding capacity and chemical compositions of yogurts were monitored at 1, 2, 3, 4, 5, 6, 7, 14 and 21 days intervals.

### Enumeration of Viable Cells in Yogurt

The enumeration of *S. thermophilus* and *Lb. delbrueckii* subsp. *bulgaricus* viable cells in yogurt were obtained by spread plating 200 μl of 10-fold serial dilutions on agar plates. For selective enumeration of *S. thermophilus*, serial dilutions were plated onto LM17 and incubated at 42°C. Selective enumeration of *Lb. delbrueckii* subsp. *bulgaricus* was performed on MRS agar plates incubated at 42°C under anaerobic conditions. *Lb. bulgaricus* and *S. thermophilus* were identified by their rough-shaped and smooth-shaped colonies, respectively. After incubation for 48 h, visible colonies were counted and expressed as colony forming units per milliliter (cfu/ml).

### Rheological Studies in the Yogurt

The viscosity of yogurt was evaluated at the end of the fermentation period using an AR2000ex Rheometer (TA Instruments, Crawley, UK) fitted with a 60 mm diameter aluminum parallel plate measurement system. The measuring geometry had a gap size of 800 μm. All measurements were made at 20°C. Samples were initially stirred to achieve a homogenous mixture. Samples were pre-sheared at 200 s^-1^ for 1 min, sheared from 0.01 to 200 s^-1^ over 2 min, held at 200 s^-1^ for 1 min and sheared from 200 to 0.01 s^-1^ over 2 min. The test was performed in triplicate.

### Water-Holding Capacity

The water-holding capacity (WHC) of the yogurt product was measured according to the method of Sodini et al. (2005). Before each analysis, yogurt samples were gently stirred to ensure homogeneity. A sample of 20 g of yogurt (*Y*) was centrifuged for 10 min at 1250 × *g* at 4°C. The whey expelled (*W*) was removed and weighed. The WHC (% w/w) was calculated as: WHC = 100*(Y*-*W)/Y*. The measurement was carried out in triplicate.

### Chemical Analysis of Yogurt

The nitrogen content of yogurt was determined by the Kjeldahl method (ISO, 2001). A nitrogen conversion factor of 6.38 was used for crude protein determination. Fat content was determined by the Röse-Gottlieb method (IDF, 1996). Moisture and total solids were determined by drying each yogurt sample for 5 h in an oven at 105°C (American Dairy Products Institute, 1990). Ash content was determined by drying at 550°C according to Horwitz (2000). Sugar content was calculated by difference [*total solids* - (*total protein* + *fat* + *ash*)] as described by Guzman-Gonzalez et al. (1999). All measurements were performed in triplicate.

### Statistical Analysis

The Student *t*-test was used to examine differences between groups. Significance was set at *p* < 0.001.

## Results

### Fish Screening

The strain isolated after plating serial dilutions of fish intestinal content was screened for GABA production in liquid LM17 broth supplemented with MSG. The strain produced over 50 μg/ml of GABA.

This GABA-producing strain was then identified using molecular techniques based on the sequencing of the 16S rRNA gene previously amplified with PCR primers pA (AGAGTTTGATCCTGGCTCAG) and pH’ (AAGGAGGTGATCCAGCCGCA) (Edwards et al., 1989). Sequencing of the resulting 1.5 kb DNA fragment and comparison with the public databases revealed a 99% identity to *S. thermophilus* strains. Hence, this strain was named *S. thermophilus* APC151 and selected for further studies.

### GABA-Production in Culture Media and Milk

In order to determine maximum rate of GABA production, *S. thermophilus* APC151 was incubated for 72 h in LM17 broth supplemented with 10 mg/ml MSG. The final concentration measured in the supernatant was 2.1 ± 0.2 mg GABA/ml culture.

To test the ability of *S. thermophilus* APC151 to synthesize GABA during milk fermentation, the strain was cultivated in skim milk supplemented with 10 mg/ml MSG. Following 72 h incubation, a similar maximum concentration of GABA (2.2 ± 0.2 mg/ml coagulated milk) was reached, thereby indicating that the switch from culture media to milk does not alter GABA biosynthesis by *S. thermophilus* APC151. In order to prevent accumulation of high levels of remaining MSG in yogurt, a second test was performed in milk fermentations supplemented with the minimum required concentration of MSG (2.25 mg/ml). Maximum GABA accumulation (2.10 ± 0.16 mg/ml coagulated milk) was in the same range indicating that 2.25 mg/ml was the minimum concentration required to reach the maximum GABA level in milk. Therefore, this concentration was selected for subsequent yogurt trials.

Under similar conditions of study, strains *S. thermophilus* CH1 and *Lb. bulgaricus* CH1 isolated from the commercial yogurt starter did not produce GABA.

### Growth Dynamics of *S. thermophilus* Strains during Yogurt Fermentation

The performance of the GABA-producer *S. thermophilus* APC151 strain during milk fermentation was investigated during fermentation of two different yogurts, the GABA yogurt (made with strains *S. thermophilus* APC151 and *Lb. bulgaricus* CH1) and the control yogurt (made with strains *S. thermophilus* CH1 and *Lb. bulgaricus* CH1). Species-specific bacterial counts were monitored over 3 weeks (which included yogurt fermentation and storage).

In both yogurt fermentations (GABA-enriched and control), *Lb. bulgaricus* CH1 viable cell counts were identical, reaching 7.8 log unit after 6 h of fermentation and these numbers remained constant until the end of the 1st week of storage. After this point viable counts started to decrease to 5 log units by the end of the 3rd week.

**FIGURE 1:**
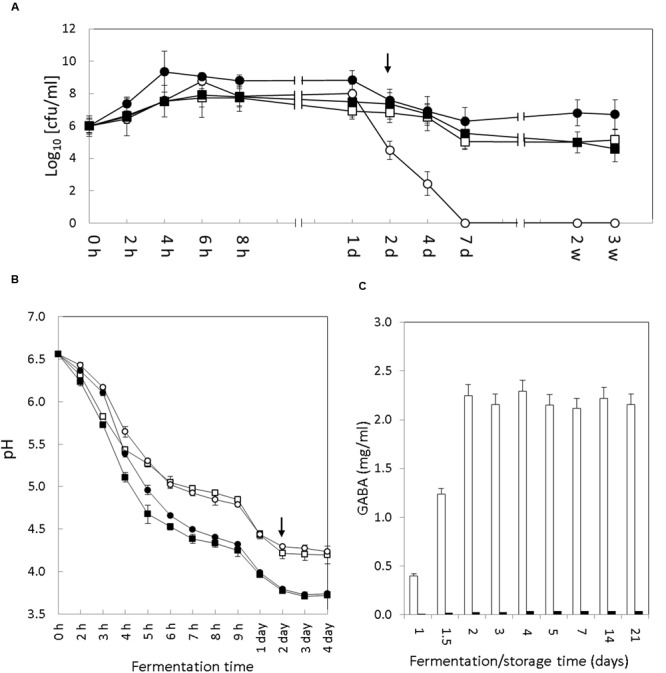
**Evolution of bacterial growth, pH and GABA accumulation in yogurt. (A)** Growth of *S. thermophilus* (*circles*) and *Lb. bulgaricus* (*squares*) cell count (cfu/ml) during fermentation and storage of the control (*empty symbols*) and GABA-enriched (*filled symbols*) yogurts. In the *Y*-axis the 0 value means <10 cfu/ml (the detection limit). **(B)** Acidification curve of *S. thermophilus* APC151 (*circles*) and CH1 (*squares*) during milk fermentation in pure monoculture (*empty symbols*) or co-cultures with *Lb. bulgaricus* CH1 (*filled symbols*). **(C)** Evolution of GABA accumulation in the GABA-enriched (*white bars*) and control (*black bars*) yogurts over fermentation and storage time. Transition between fermentation and storage is indicated by the arrow [h, hours; d, days; w, weeks]. The representation of time on *X*-axis is not to scale.

The colony counts of both *S. thermophilus* strains increased exponentially during the initial hours of fermentation to reach a maximum of 9 log units (**Figure 1A**). This maximum was reached in 4 h of fermentation by the APC151 strain (μ_max_ = 0.16 ± 0.01) and in 6 h of fermentation by the control CH1 strain (μ_max_ = 0.13 ± 0.01). Following the first 24 h, the viability of the *S. thermophilus* strains started to decrease. Noticeably, although this effect was observed in both strains (APC151 and CH1), the reduction was more severe in the industrially used CH1 strain (4.5 log units after 48 h and <1 log units after day 7) than in the APC151 strain (7.5 log units after 48 h and 6.3 log units after day 7).

Interestingly, the performance of the two starters was different in the two yogurts. In CH1 yogurt the *S. thermophilus* outnumbered *Lb. bulgaricus* (9 versus 7.8 log units) over the first 24 h but this was reversed after the 2nd day. In contrast, the *S. thermophilus* APC151 strain was the dominant species in the GABA-enriched yogurt during the fermentation and storage and remained viable (6.7 log units) after 3 weeks of storage at 4°C.

### Acidifying Kinetics of *S. thermophilus* Strains during Milk Fermentation

The rate of milk acidification by *S. thermophilus* is a technological trait of major importance for yogurt manufacture. Hence, the pH curves of the control and GABA yogurts were recorded during the fermentation process (2 days) and the initial 2 days of storage. Interestingly, the acidification curves monitored in both yogurts were very similar (**Figure 1B**). Although initial pH decrease was slightly faster in the control yogurt, both yogurts reached a pH value below 5.0 after 5 h, and by 8 h of fermentation the pH was 4.4; thereby defining an acidification rate (ΔpH/Δ*t*) of 0.33 ± 0.02 h^-1^ for the control CH1 yogurt and 0.31 ± 0.02 h^-1^ for the GABA-enriched yogurt. The yogurt pH at the end of the fermentation (48 h) was 3.8 and after this time this value remained stable during storage at 4°C for 3 weeks.

Since both *S. thermophilus* strains were able to ferment milk individually (without co-culturing with *Lb. bulgaricus*), we also characterized the pH curve of each strain in milk. Again, pH curves were analogous for both strains CH1 and APC151 (**Figure 1B**). Compared to the co-culture with *Lb. bulgaricus*, the pH decrease was less intensive, since pH 5.0 was reached after 6 h, and by 8 h pH was 4.8 (ΔpH/Δ*t* = 0.22 ± 0.02 h^-1^). The milk pH at the end of the fermentation (48 h) was 4.28 and after this time this value remained stable during storage at 4°C.

### GABA Production during Yogurt Fermentation

The presence of GABA in the control and GABA yogurts was tested at different timepoints during yogurt fermentation and storage (1, 1.5, 2, 3, 4, 5, 7, 14, and 21 days) (**Figure 1C**). There was no production of GABA detected in the control yogurt. However, in the GABA yogurt, accumulation of GABA started after the 1st day and reached the maximum concentration (2.2 mg/ml) by the end of the fermentation process (2 days). After this time, GABA concentration remained constant until the end of the storage period (3 weeks) at 4°C.

### Viscosity of the GABA Yogurt

**FIGURE 2:**
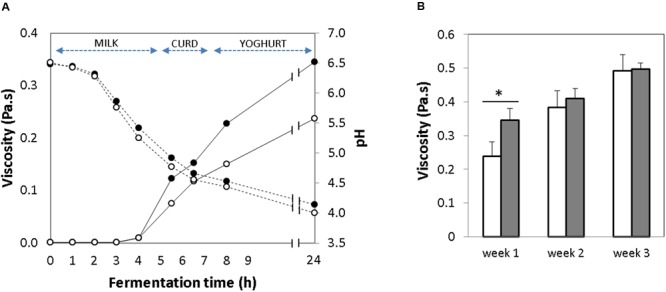
**(A)** Change in viscosity (*solid line*) and pH curve (*dotted line*) over milk fermentation during 24 h [*Empty circles:* control yogurt; *Filled circles:* GABA-enriched yogurt]. **(B)** Evolution of viscosity over storage time for 1, 2, and 3 weeks [GABA-enriched (*filled bars*) and control (*empty bars*)]. ^∗^*p*-value < 0.001.

The acidification that occurs during milk fermentation results in milk coagulation due to destabilization of the micellar casein. Thus, yogurt viscosity is an indication of gelation (Nouri et al., 2011). The evolution of the viscosity was evaluated in the GABA-containing and the control yogurts during the initial 24 h of the fermentation. In both yogurts, the viscosity started to increase gradually after 5 h of fermentation, just when the pH decreased below 5.0, and a complete coagulation occurred 2 h later, when the pH was 4.5 (**Figure 2A**). After the 1st week of storage, the viscosity of the GABA yogurt (0.34 ± 0.04 Pa-s) was significantly higher (*p*-value < 0.005) than that of the control yogurt (0.23 ± 0.05 Pa-s) (**Figure 2B**). After this time, viscosity of the control and GABA yogurts increased gradually during storage for 2 (0.38 ± 0.04 and 0.40 ± 0.03 Pa-s, respectively) and 3 (0.49 ± 0.04 and 0.49 ± 0.01 Pa-s, respectively) weeks. Thus, at the end of the storage period, the viscosity of both yogurts was not significantly different.

### Syneresis of the GABA Yogurt

**FIGURE 3:**
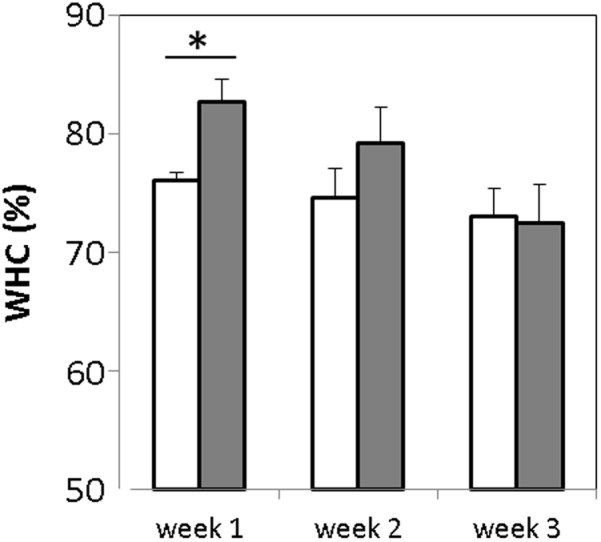
**Measurement of water holding capacity of GABA-enriched (*filled bars*) and control (*empty bars*) yogurts after storage for 1, 2, and 3 weeks.** ^∗^*p*-value < 0.001.

Syneresis is known to have an important impact on quality and acceptability of some fresh fermented products such as yogurt. The water holding capacity (WHC) of the GABA-containing and the control yogurts was evaluated once a week during the storage period. The WHC values obtained were 76.02 ± 1.05 and 82.71 ± 1.8% for the control and GABA-containing yogurts, respectively (**Figure 3**). Hence, the latter has a slightly but significantly lower (*p*-value < 0.005) susceptibility to syneresis when compared to the control yogurt. After the 1st week, the WHC of the GABA yogurt decreased gradually, whereas that of the control yogurt held steady, thereby the WHC of both yogurts was similar after the 2nd week of storage.

### Chemical Composition

The GABA yogurt was evaluated for chemical properties using the existing commercial CH1 yogurt as control. The results (data not shown) demonstrate that the chemical composition of the GABA-containing and reference yogurts was comparable [moisture (87–88%), protein (5.05–5.65%), fat (0.12–0.15%), sugar (4.8–5.8%), and ash content (0.74–1.2%)].

Yogurt composition did not vary over the storage period of 3 weeks. Thus, there was no significant difference in the chemical composition of the GABA-enriched and control yogurts.

## Discussion

In this work, we isolated and identified a *S. thermophilus* strain able to produce GABA during milk fermentation and demonstrated its performance and suitability to be used as a starter strain for yogurt manufacture. Prior to storage, the fermentation was carried out for 48 h, the minimum time required to reach maximum GABA production levels. Despite the prolonged fermentation time, no adverse characteristics in the yogurt were observed; viscosity and consistency increased and no whey syneresis was observed. Regarding acidity, the final pH (4.3) was in the range expected for yogurt (typically between 4 and 5) (Lund et al., 2000).

The use of the *S. thermophilus* APC151 as a starter GABA producer strain developed in this work would yield a yogurt containing 2 mg/ml GABA. The ingestion of one unit of this yogurt (100 g) would provide the consumer with 200 mg of GABA. This concentration is above the bioactivity threshold previously reported to result in blood-pressure-lowering and antidiabetic effects on the consumer (Inoue et al., 2003; Pouliot-Mathieu et al., 2013; Wan et al., 2015). Daily ingestion of 100 ml of the GABA yogurt obtained in this study would supply a total of 200 mg GABA, a dose equivalent to the GABA content of a tablet (the daily recommended serving) of PharmaGABA^TM^, which is documented to have health benefits (Food and Drug Administration [FDA], 2008).

Other fermented products enriched in GABA using GABA-producing LAB as starters have been developed. These include black raspberry juice (using *Lb. brevis* as source of GABA) (Kim et al., 2009), kimchi (using *Lactobacillus sp.* as source of GABA) (Seok et al., 2008), sourdough (*Lb. plantarum* and *L. lactis* as source of GABA) (Rizzello et al., 2008), fermented soya milk (*Lb. brevis* as source of GABA) (Park and Oh, 2007), and cheese (*L. lactis* as source of GABA) (Nomura et al., 1998; Pouliot-Mathieu et al., 2013). The development of GABA-containing ‘so-called’ yogurt has also been pursued using *Lb. plantarum* as biological source of GABA (Shan et al., 2015). However, this product cannot be called yogurt anymore in most countries due to legal issues (defined on a national law). The species typically associated with the technological production of the yogurt are *S. thermophilus* and *L. delbrueckii* subsp. *bulgaricus* (Han et al., 2014). Recently, a *S. thermophilus* strain was utilized to ferment and enrich milk in GABA (Chen et al., 2016). However, the biotechnological attributes of this strain for yogurt production were not studied and the fermentation was not tested in co-culture with *L. bulgaricus*, as usually occurs in yogurt manufacture. In this work, we isolated a *S. thermophilus* strain technologically suitable for the production of a yogurt containing 200 mg of GABA per serving without affecting its microbiological, physical, and chemical properties. In this regard, strain APC151 showed a milk acidity curve comparable to that of a strain used as commercial starter for yogurt manufacture. *S. thermophilus* strains in monoculture decreased milk pH to 4.2. A lower value (3.75) was reached when co-cultured with the *Lb. bulgaricus* strain. This additional pH decrease is caused by the *Lb. bulgaricus* strain, which is less susceptible to acid, thereby gradually dominates the overall fermentation (Lourens-Hattingh and Viljoen, 2001; Sfakianakis and Tzia, 2014), as we observed in the control yogurt. However, in the GABA yogurt the *S. thermophilus* strain remained viable throughout yogurt shelf life, which could be due to a higher acid tolerance attributable to its capability to decarboxylate glutamate and produce GABA and ammonia. This fact could provide the strain an advantage to survive the low pH conditions of the intestine. This ability is one of the basic requirements for a microbe to produce GABA in the intestine (Bravo et al., 2011).

During the 1st week of storage, the yogurt produced by the GABA-producer strain had a higher viscosity thereby suggesting a higher yogurt body, thickness, firmness, and consistency. This could be due to the fact that the CH1 thermophilic starter culture is intended to produce yogurt with strong flavor and low viscosity (CH-1 Yo-Flex^®^ Specifications, Christian Hansen, Denmark). Nevertheless, after this time viscosity of both yogurts was not significantly different. A slight decrease of the WHC over the storage period was observed in the two yogurts, which may be correlated with higher syneresis. Long storage periods can cause alteration of the yogurt microstructure, which ultimately leads to syneresis (Mao et al., 2001; Aryana et al., 2006; Nguyen et al., 2015).

In conclusion, *S. thermophilus* APC151 strain can ferment milk and yield yogurt with equivalent microbial, rheological and chemical properties to those observed in yogurt obtained with a *S. thermophilus* strain typically used in industry. The antibiotic resistance pattern of this strain was profiled and atypical resistances were not found (data not shown). The *S. thermophilus* strain proposed here provides GABA enrichment in the yogurt, offering the possibility of production of new naturally fermented GABA fortified yogurt with an added value.

## Author Contributions

DML provided the general concept, designed and performed the work, analyzed and interpreted the results and drafted the manuscript. TOC and POC performed some experiments and revised the manuscript. CS and RPR contributed to the discussion of the research and revised and approved the manuscript.

## Funding

This work was funded by the APC Microbiome Institute, a Centre for Science and Technology (CSET) funded by the Science Foundation Ireland (SFI), through the Irish Government’s National Development Plan.

## Conflict of Interest Statement

The authors declare that the research was conducted in the absence of any commercial or financial relationships that could be construed as a potential conflict of interest.
